# Volcaniclastic tide-modulated tempestite in a coastal tuff ring, Jeju Island, Korea

**DOI:** 10.1038/s41598-019-39784-0

**Published:** 2019-03-05

**Authors:** Chanwoo Sohn, Young Kwan Sohn

**Affiliations:** Department of Geology and Research Institute of Natural Science, Gyeongsang National University, Jinju, 52828 Republic of Korea

## Abstract

The need for more accurate and extensive records of storm activity is increasing because of growing population and infrastructure in coastal areas. Records of past storm activity have consequently been sought from diverse sedimentary proxies in marginal marine environments, as well as historical documents. In this paper, we introduce a unique record of a 3.7 ka BP storm event preserved in a coastal tuff ring on Jeju Island, Korea. The tuff ring formed mostly above high tide level by primary volcanic processes, but contains three interbeds of horizontally laminated to hummocky/swaly cross-stratified deposits with intervening mud drapes up to an altitude of ~4.6 m above high tide level. These interbeds were formed by wave activity in a swash to surf zone when the sea level rose several meters above normal high-tide level during a storm event, and the triple intercalation of the wave-worked deposits reflects three tidal cycles during a storm event that lasted 1.5 day. Jeju Island contains the most complete record of an ancient storm event ever reported in the tuff ring, and highlights the significance of coastal volcanoes in the study of extreme depositional events in coastal areas, such as storms and tsunamis.

## Introduction

Volcaniclastic deposits can preserve high-resolution records of earth surface processes, such as daily to millennial-scale sea-level fluctuations^1,2^ and fair-weather marine processes^3^, even in coastal areas vulnerable to erosion because they accumulate commonly with extremely high sedimentation rate. In this paper, we introduce a unique volcaniclastic deposit from Jeju Island, Korea, which we interpret as having resulted from a storm event during the eruption of a coastal phreatomagmatic volcano, named the Songaksan tuff ring, which was produced by explosive magma-water interaction in the subsurface^4^. Here we show that the tuff ring was partly submerged and wave-worked during the storm event, and could preserve the most complete record of an ancient storm event ever reported because of extremely rapid deposition of tephra supplied from a contemporaneous volcanic eruption. Based on detailed sedimentological analysis of the deposit, we reconstruct a hybrid depositional event that happened by fortuitous combination of a storm and a volcanic eruption that are superimposed on daily tide. This finding has significant implications regarding the interpretation of depositional records of extreme events in coastal environments such as storms and tsunamis.

## Site Characteristics

**Figure 1:**
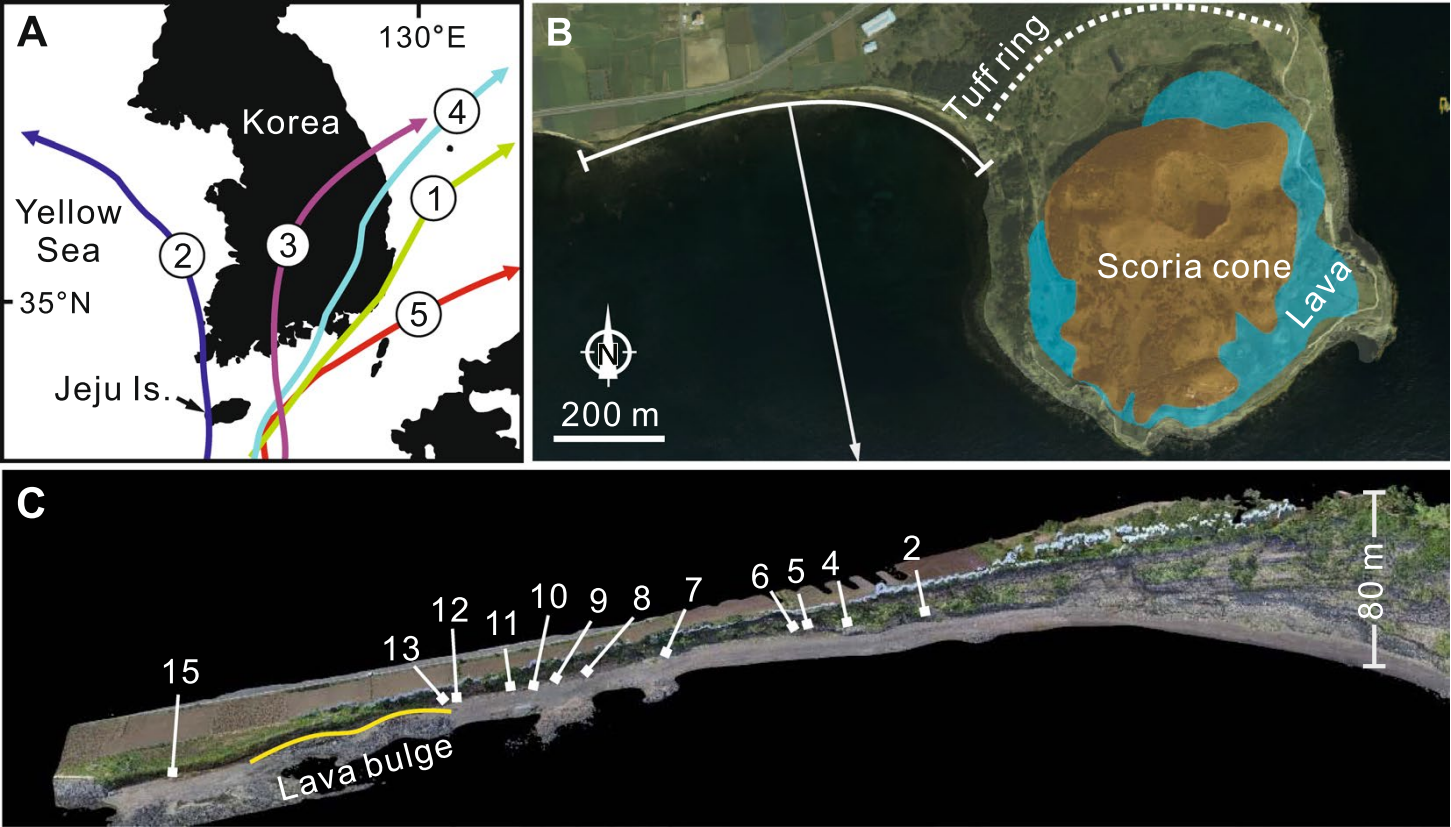
Location of study area. (**A**) Location of Jeju Island. Tracks of the five largest typhoons that hit the Korean Peninsula in the last half century are indicated. 1 = Sarah, 1959, 2 = Rita, 1972, 3 = Rusa, 2002, 4 = Maemi, 2003, 5 = Chaba, 2016. (**B**) Geometrically corrected aerial photograph (orthophotograph) of Songaksan, taken by the authors in 2015, consisting of a tuff ring, a nested scoria cone and a ponded lava inside the crater of the tuff ring. The crater rim of the tuff ring is indicated by a dashed line. (**C**) 3D model of the exposure of the tuff ring along the western coast with the locations of measured localities, which was built by combining hundreds of still images taken with a drone.

Jeju Island is an alkali basaltic volcano built in a tectonically stable intraplate setting in the southeastern Yellow Sea^5^ (Fig. 1A). The island is composed of shield-forming lavas and hundreds of small volcanic cones that have formed throughout the Quaternary. Songaksan is the youngest volcanic center on the island, which erupted ~3.7 ka BP^1,6^ and consists of a basaltic tuff ring, a nested scoria cone and a ponded lava inside the crater^1^ (Fig. 1B). The entire tuff ring resulted from a single continuous eruption^4^ possibly in a month^2^. The sea level at that time was almost identical to that at present. Paleo-high tide level on a day of the eruption, inferred from the intertidal to supratidal facies transition in the tuff sequence^2^, is 0.8 m, deviating very little from the mean high tide level of 1 m at present, considering the diurnal inequality of the tide. (All altitude data were obtained by the South S82T RTK GPS surveying unit and are expressed in terms of mean sea level.) Vertical crustal motion is regarded to have been negligible in the Jeju Island area because the area is located in an intraplate setting that is tectonically more stable than other regions in East Asia^7^. The tide is semidiurnal, and the tidal range is 1.7 m. Typhoons make landfall 3.1 times a year (averaged over 107 years), mainly between July and September^8^. Most typhoon routes are adjacent to Jeju Island (Fig. 1A).

## Deposit Characteristics

**Figure 2:**
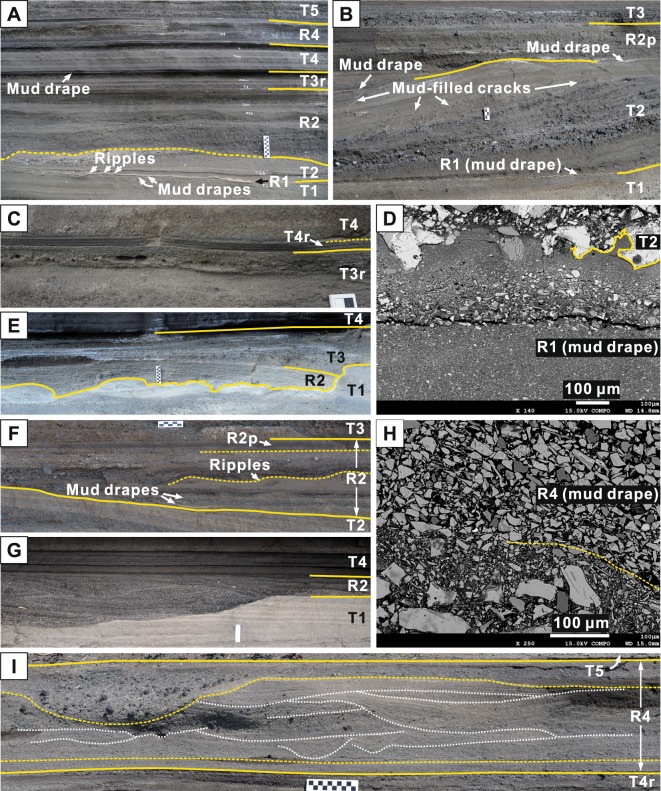
Outcrop photographs and thin-section images. (**A**) An exposure at locality 11 showing a complete sequence of reworked units (R1 to R4) and interbedded primary tuff units (T1 to T5). Unit T3 here is thinly stratified and overlain by a mud drape. It is interpreted to be reworked and is distinguished by a subscript ‘r’ at the end of the unit name. (**B**) Megaripple-bedded tuff (unit T2) at locality 5, underlain by a mud drape (unit R1) and planar-bedded tuff (unit T1). Unit R2 here is a poorly sorted, crudely stratified, primary tuff, and is distinguished by a subscript ‘p’ at the end of the unit name. (**C**) The basal 1 cm of the airfall tuff unit T4 at locality 9 is horizontally laminated due to reworking by swash waves, and is distinguished by a subscript ‘r’ at the end of the unit name. (**D**) Back-scattered electron image of the mud drape of unit R1 at locality 11. (**E**) Decimeter-deep erosion beneath the low-angle cross-stratified unit R2 at locality 8. Unit T2 is completely removed by erosion. (**F**) Unit R2 at locality 6 shows a vertical facies change from multiple mud drapes at the base, seaward-migrating ripple formset and high-angle cross-stratification in the middle, well-sorted and horizontally laminated deposits above it, to poorly sorted and crudely stratified primary tuff (R2p). (**G**) Swaly cross-stratified deposit of unit R2 at locality 15, filling a decimeter-deep scour. Unit T2 is completely removed by erosion probably together with unit R1 and part of unit T1. (**H**) Back-scattered electron image of a mud drape of unit R4 at locality 9, composed of well-sorted and imbricated silt-size ash. The lower part of the image, also belonging to unit R4, is much coarser-grained and poorer sorted. (**I**) Hummocky to swaly cross-stratified division of unit R4 at locality 10 sandwiched between horizontally laminated divisions at the base and at the top. The upper division passes laterally into a massive and coarse-grained deposit filling an ebb-related scour hollow. All photo scales are graduated in centimeters.

The Songaksan tuff ring accumulated mostly above high tide level by pyroclastic surges and airfall except for the basal one meter-thick section, which was deposited in an intertidal setting and contains wave-worked deposits^2^. In the medial to distal tuff ring (Fig. 1C), another set of ‘apparently reworked’ deposits is intercalated in the middle of the tuff sequence, for which we defined three reworked units (R1, R2, and R4) and five primary tuff units (T1 to T5) (Fig. 2A). We use the term ‘reworked’ in a tentative way as some of the ‘apparently reworked’ deposits may be primary in origin, with tephra particles derived directly from the vent, without involving interim storage prior to arrival at the final depositional site^9^.

**Figure 3:**
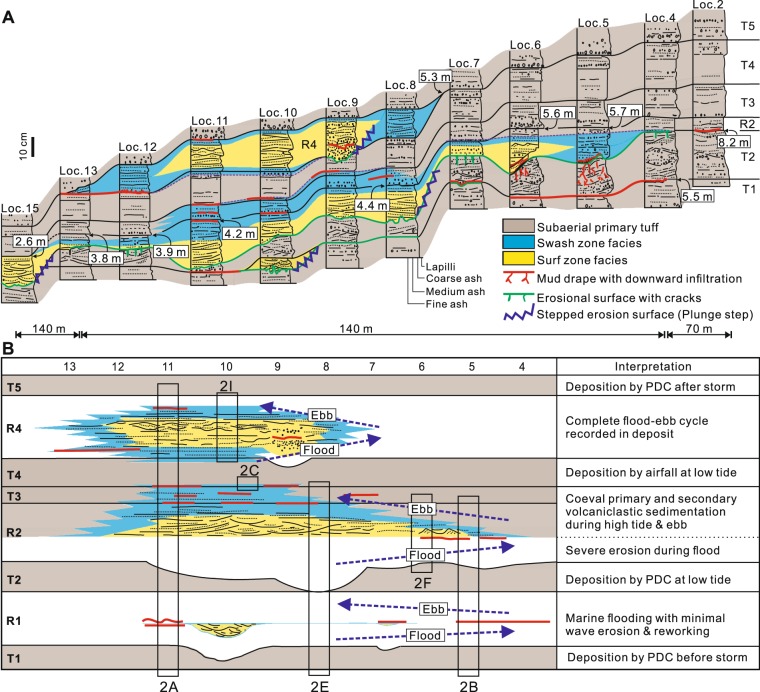
Graphic columns and chronostratigraphic chart. (**A**) Correlated columns, showing three interbeds of wave-worked deposits between primary tuff beds. (**B**) Chronostratigraphic illustration of the primary and reworked deposits, reflecting three episodes of sea-level rise and fall due to tidal fluctuations. Approximate locations of the photos in Fig. 2 are indicated by boxes. PDC = pyroclastic density current.

The primary tuff units are generally poorly sorted and crudely to thinly and planar- to undulatory-stratified (units T1, T3, and T5) or climbing megaripple-bedded (unit T2) (Fig. 2A,B), suggesting emplacement by pyroclastic surges^10^. Unit T4 is characterized by diffuse but continuous stratification with abundant accretionary lapilli (Fig. 2A), suggesting emplacement by airfall. All these units thicken and coarsen towards the crater rim. Unit T3 is generally poorly sorted and crudely stratified (e.g., Fig. 2B) but passes laterally into a better-sorted, low-angle stratified deposit, overlain by a mud drape between localities 8 and 11 (Fig. 3A). The basal and topmost few centimeters of unit T4 are also well-sorted and horizontally laminated at these localities (Fig. 2C), suggesting local reworking of these units.

Unit R1 consists of a trough cross-stratified deposit filling a decimeter-deep scour hollow at locality 10, and comprises a single, mm-thick mud drape between localities 4 and 7 (Fig. 2B), extending up to an altitude of 5.5 m (Fig. 3A). At locality 11, the unit includes two mud drapes that encase a very fine sand/silt layer with a rippled upper surface (Fig. 2A). The mud is composed of mainly nonvolcanic materials (illite, quartz, and biotite) in addition to fine glass shards and plagioclase. The mud layer is either massive or variably graded and loaded by the overlying deposit (Fig. 2D).

Unit R2 shows a prominent erosion surface at the base throughout the measured localities (Fig. 3A), part of which was carved in the form of elongate scours aligned in the SW-NE direction (Fig. 2E). The erosion surface is overlain by discontinuous mud laminae in the troughs of the underlying megaripple bedforms up to an altitude of 8.2 m (Figs 2F, 3A). The underlying unit T2 shows deformation structures that formed probably during deposition of unit R2, including (1) a landward-overturned convolute structure and (2) numerous vertical to dendritic cracks, some of which were infiltrated by mud from the overlying mud drape (Fig. 2B). The internal structure of unit R2 is dominated by horizontal to very low-angle or laterally pinching-out stratification in the upper part, and by either landward- or seaward-dipping, low- to high-angle and swaly cross-stratification in the lower part (Fig. 2E,G) with local seaward-migrating ripple formset (Fig. 2F). Unit R2 passes laterally into poorly sorted, crudely stratified, primary tuff towards the crater rim (Fig. 3A).

Unit R4 occurs up to an altitude of 5.3 m, and is characterized by an overall lack of erosion at the base (Fig. 3A), and an abundance of hummocky to swaly cross-stratification, under- and overlain by horizontally laminated divisions (Fig. 2I). Mud drapes are locally preserved at the base and top of the unit, commonly in association with horizontally laminated deposits (Fig. 3A). The mud drapes are composed of well-sorted, imbricated, coarse silt-size ash (Fig. 2H).

## Interpretation

The reworking processes that are responsible for units R1 to R4 and part of units T3 and T4 are interpreted to be marine because the reworked facies occur exclusively below ~5.6 m (except for a mud drape at loc. 2, Fig. 3A). Reworking by rainfall-induced surface runoff is negated by the common occurrence of landward-dipping cross-strata in the reworked deposits. Considering the duration of phreatomagmatic eruptions, which commonly last weeks to months^11^, and the inferred eruption duration of Songaksan, which could have been as short as a month^2^, the reworking is interpreted to have taken place within days. Reworking by a tsunami over a few hours might be an alternative interpretation, but this possibility is discarded because of the prevalence of wave-formed structures in the reworked deposits. The reworking is therefore attributed to a storm surge event that temporarily inundated the tuff ring for a day or two.

The submerged portion of the tuff ring is inferred to have had a smooth, concave-up profile that is featureless except for an ~1 m-high lava bulge between localities 13 and 15 (Fig. 1C), toward which reworked deposits pinch out. The slope of the submerged tuff ring averages 0.020 between localities 2 and 13, and increases from 0.010 to 0.025 toward the crater rim. The Iribarren number, a dimensionless parameter used to model the effects of surface gravity waves on beaches^12^, of the submerged tuff ring is estimated to be 0.1 when the extreme values of wave parameters observed during major typhoons in Korea are used, i.e., a deep-water significant wave height *H*_*o*_ of 12 m and a wave period of 15 s. The Iribarren number is sufficiently small to make the incident waves dissipate much of their energy by spilling before reaching the shoreline^12^ such that the majority of the submerged tuff ring was within the surf zone during the storm.

The 2% exceedence of storm-induced runup *R*_2_ caused by both incident and infragravity waves was estimated using an empirical formula^13^, which can be applied to a dissipative beach with a small Iribarren number (<0.3):

1 $${R}_{2}={0.043}^{2}\sqrt{{H}_{o}{L}_{o}}$$

where *L*_*o*_ is the deep-water wavelength defined as *gT*2*/*2*π*. For extreme wave conditions (*H*_*o*_ = 12 m; *L*_*o*_ = 350 m when *T* is 15 s), the wave runup is calculated to be 2.8 m. Considering the average tidal fluctuations (high tide level of 1 m and low tide level of −0.7 m) and the maximum observed storm surge height of 1 m, the tuff ring could have been inundated up to an altitude of 4.8 m at high tide and 3.1 m at low tide. The observed occurrence of the swash zone facies in unit R2 (~5.6 m) and unit R4 (~5.3 m) above the estimated runup suggests that the wave conditions were more extreme, or that there was local amplification of runup because of unknown coastal conditions. The estimated runup sea level at low tide coincides well with the level of the transition between the swash zone and the surf zone facies of unit R2 at locality 15 (Fig. 3A).

The horizontally to low-angle stratified deposits are interpreted to have formed by upper plane bed deposition on a beach face by swash and backwash of breaking waves^14^. The low- to high-angle cross-stratification is interpreted to have formed by either seaward or landward migration of bedforms within the transition between surf and swash or within the inner portion of the surf zone^15^. The hummocky to swaly cross-stratification was produced most likely by wave-induced combined flows in the surf zone^16^. Local seaward-migrating ripple formsets (Fig. 2A,F) are attributed to a return or relaxation flow in the surf zone driven by coastal setup. The transition between the swash zone and the surf zone facies is commonly characterized by decimeter-deep ‘stepped’ erosion at the base (Figs 2E, 3A), probably representing a plunge step which develops immediately seaward from the base of the beach. The transition is therefore interpreted to indicate the approximate position of the shoreline, either at high tide or low tide.

The fine-grained mud drape of unit R1 (Fig. 2D) suggests suspension settling of fines in still or weakly wave-agitated water. The mud drapes of units R2 and R4, which are characterized by good sorting and imbrication of grains (Fig. 2H) and occur in close association with the swash zone facies (Fig. 3), suggest deposition of fine ash in a swash zone under a weak wave-generated motion of the water. We presume that the shoreline was hit by mostly infragravity waves, of which the dominant period is 80 to 300 s^17^, and that there were chances for the suspended fines to settle on the bed between the strokes of the waves, even in the swash zone.

Intercalation of multiple wave-worked deposits within the primary tuff beds and the landward or seaward translation of the shoreline, as depicted in the chronostratigraphic chart (Fig. 3B), indicate that there were fluctuations of sea level during the storm event. Vertical facies variations within individual units also suggest changing sea levels during deposition of the reworked units. In unit R2, seaward-migrating ripple formset passes upward into horizontally laminated deposits and into primary tuff (Fig. 2F), suggesting gradual emergence of a surf zone to a subaerial surface. In unit R4, hummocky to swaly cross-stratified deposit is sandwiched between horizontally laminated deposits (Fig. 2I), indicating deepening and shallowing of the depositional site from swash zone to surf zone and then to swash zone. These sea-level fluctuations were most likely due to tides superimposed on a storm surge. Three consecutive storm events appear improbable. We thus interpret that the triple intercalation of wave-worked deposits in Songaksan reflects three tidal cycles during a ~1.5 day-long storm event.

Changes in the character of the reworked units suggest further that there was waxing and waning of wave intensity during the storm event. Unit R1, characterized by the absence of swash zone facies and the localized reworking only at locality 10 (Fig. 3A), suggests inundation of the tuff ring up to an altitude of ~5.5 m followed by suspension settling of fines without significant wave activity throughout the first tidal cycle of the storm event. On the other hand, wave erosion was severe during the flood of the second tidal cycle, resulting in the prominent erosion surface beneath unit R2 and the deformation structures in unit T2, which suggest strong shear and normal stresses on the seabed provided by strong pounding waves.

Volcanic eruption occurred during deposition of unit R2, resulting in primary tuff deposition in proximal localities (Figs 2F, 3A). The eruption was immediately followed by the eruption of units T3 and T4, which were thus partly reworked by swash during the ebb of the second tidal cycle. The eruption of unit T4 continued until the onset of the next flood. The topmost part of unit T4 was thus reworked by swash during the flood of the third tidal cycle. Wave activity was moderate during deposition of unit R4, leaving a complete deposit record of a flood-ebb cycle (Figs 2I, 3B).

## Implications

This study demonstrates that volcaniclastic deposits in coastal phreatomagmatic volcanoes can preserve the record of past storm events in an unusual detail that is almost never provided by ordinary sedimentary deposits because of their extremely rapid sedimentation and burial by later deposits, preventing post-depositional erosion, reworking or bioturbation. This finding therefore highlights the potential significance of coastal phreatomagmatic volcanoes in the study of paleotempestology^18^ because these volcanoes are second only to scoria cones in abundance on Earth, common in coastal areas, and can be affected by storms in regions of frequent storms in spite of their short eruption duration. Coastal volcanoes therefore seem to represent a potential source of high-resolution proxy records of past storm activity, which have been overlooked to date, but are worthy of close examination in the future.

This study demonstrates that the tide can play an important role in determining the stratal characteristics of storm deposits because the tidal range can be as large as the wave runup and the storm surge height, even in a microtidal setting. The possibility of generation of multiple tempestite beds during a single storm event because of tidal fluctuations even in a microtidal setting also provides significant implications regarding the interpretation of paleotempest deposits in marginal marine environments. Common intercalation of mud drapes in the storm deposits in Songaksan also raises a problem regarding the distinction between storm deposits and tsunami deposits because such mud laminae have not been reported in storm deposits, and have been regarded to be a characteristic feature of tsunami deposits^19^. A recent study, suggesting that the coastal setup can oscillate with the incidence of large and small wave groups and can steepen into a tsunami-like wave^20^, further complicates this issue. Further investigation of the role of long-period waves such as infragravity waves or surf beats thus seems necessary for a better understanding of extreme depositional events in coastal regions and how they are imprinted in sedimentary proxy records.

## Methods

### Sedimentological Observations

Detailed cm-scale (1:10) measurements of sedimentological properties of the deposits were made at twelve sites along the western shore of the Songaksan tuff ring. Sedimentological properties described in the field include bed thickness, grain size, depositional and erosional structures, post-depositional deformation structures, bed geometry and lateral bed continuity, sorting, and clast shape. Correlation of individual depositional units were made by tracing each unit between these sites. The altitudes of key stratigraphic surfaces were obtained by the South S82T RTK GPS surveying unit.

### Microscopic Observations

For microscopic observations of the deposits, which are unconsolidated and fragile, undisturbed specimens were carefully sampled and packed with plastic wrapping paper in the field. They were then dried and impregnated with epoxy in the laboratory, and prepared for polished thin sections. Back-scattered electron (BSE) images were acquired using a field-emission scanning electron microscope (JEOL JSM-7610F) at Gyeongsang National University.

### Estimation of Wave Runup

To estimate the storm-induced inundation of the tuff ring quantitatively, the 2% exceedence of storm-induced runup *R*_*2*_ was estimated using an empirical formula^13^, which takes into account both incident and infragravity waves. We also estimated the Iribarren number because this formula can be applied to a dissipative beach with a small Iribarren number (<0.3). We used the extreme values of wave parameters to calculate this formula and the number, i.e., a deep-water significant wave height *H*_*o*_ of 12 m, a wave period of 15 s, and a surge height of 1 m. These values have been observed during major typhoons in Korea in the last one century by the Meteorological Administration of Korea^8^.

## Data Availability

The datasets generated during and/or analysed during the current study are available from the corresponding author on reasonable request.
